# The internal initiation of translation in bovine viral diarrhea virus RNA depends on the presence of an RNA pseudoknot upstream of the initiation codon

**DOI:** 10.1186/1743-422X-4-124

**Published:** 2007-11-22

**Authors:** Lorin Moes, Manfred Wirth

**Affiliations:** 1 Evolva, CH-4123 Allschwil, Switzerland; 2 Molecular Biotechnology, Helmholtz Centre for Infection Research HZI, D-38124 Braunschweig, Germany

## Abstract

**Background:**

Bovine viral diarrhea virus (BVDV) is the prototype representative of the pestivirus genus in the *Flaviviridae *family. It has been shown that the initiation of translation of BVDV RNA occurs by an internal ribosome entry mechanism mediated by the 5' untranslated region of the viral RNA [1]. The 5' and 3' boundaries of the IRES of the cytopathic BVDV NADL have been mapped and it has been suggested that the IRES extends into the coding of the BVDV polyprotein [2]. A putative pseudoknot structure has been recognized in the BVDV 5'UTR in close proximity to the AUG start codon. A pseudoknot structure is characteristic for flavivirus IRESes and in the case of the closely related classical swine fever virus (CSFV) and the more distantly related Hepatitis C virus (HCV) pseudoknot function in translation has been demonstrated.

**Results:**

To characterize the BVDV IRESes in detail, we studied the BVDV translational initiation by transfection of dicistronic expression plasmids into mammalian cells. A region coding for the amino terminus of the BVDV SD-1 polyprotein contributes considerably to efficient initiation of translation. The translation efficiency mediated by the IRES of BVDV strains NADL and SD-1 approximates the poliovirus type I IRES directed translation in BHK cells. Compared to the poliovirus IRES increased expression levels are mediated by the BVDV IRES of strain SD-1 in murine cell lines, while lower levels are observed in human cell lines. Site directed mutagenesis revealed that a RNA pseudoknot upstream of the initiator AUG is an important structural element for IRES function. Mutants with impaired ability to base pair in stem I or II lost their translational activity. In mutants with repaired base pairing either in stem 1 or in stem 2 full translational activity was restored. Thus, the BVDV IRES translation is dependent on the pseudoknot integrity. These features of the pestivirus IRES are reminiscent of those of the classical swine fever virus, a pestivirus, and the hepatitis C viruses, another genus of the *Flaviviridae*.

**Conclusion:**

The IRES of the non-cytopathic BVDV SD-1 strain displays features known from other pestivirus IRESes. The predicted pseudoknot in the 5'UTR of BVDV SD-1 virus represents an important structural element in BVDV translation.

## Introduction

The pestiviruses like bovine viral diarrhea virus (BVDV), classical swine fever virus (CSFV) and border disease virus (BDV) are the causative agents of economically important diseases of cattle, pigs and sheep. Due to similarities in genome organization and structure of the 5 'UTRs pestiviruses are distantly related to hepatitis C virus (HCV). Pestiviruses and hepatitis C virus are small, enveloped viruses containing single-stranded, plus-sense RNA genomes 10–12 kb in length. The mRNA contains one long open reading frame coding for a polyprotein. The coding region is preceded by a highly-structured 5' UTR of 300–400 nt in length harboring multiple AUGs which are not used for initiation of translation. Previous investigations showed that translation initiation in BVDV, CSFV and HCV occurs by an internal ribosomal entry mechanism [1-8]. The HCV internal ribosomal entry site (IRES) has been investigated in detail and the delimitation of the IRES, as well as structural pecularities, have been reported [9]. Unlike the prototype IRES elements of poliovirus or EMCV, the HCV IRES is relatively short encompassing about 300 nucleotides. Interestingly, the region immediately downstream of the initiator AUG has been found to increase translational efficiency suggesting that the IRES extends into the coding region, a feature not found in the IRES of picornaviruses [10,11]. Remarkably, the HCV IRES as well as the CSFV IRES contain a functional RNA pseudoknot structure upstream of the polyprotein initiation site that is indispensable for internal initiation of translation [3,12-14]. In contrast to the popular HCV IRES, less is known about the BVDV IRES. Hybrid arrest translation experiments, Poole et al. [1] suggested that the initiation of translation is mediated by a part of the 385 nt long 5' UTR. Dicistronic transfection experiments demonstrated that the IRES of the BVDV-NADL strain 5' UTR functions in BHK and CV1 cells. The 5' border has been mapped and the requirement of defined regions in the secondary structure of the 5' UTR have been investigated [1,2]. As a 21% reduction was observed when deleting coding sequences of the polyprotein in these experiments the IRES seems to extend into the BVDV NADL coding region [2]. However, the exact dimension of contributing coding sequences as well as the importance of the putative pseudoknot region upstream of the initiator AUG has not yet been addressed. To characterize the BVDV IRES in detail, we studied the translational initiation of BVDV strains NADL (cytopathic) and SD-1 (non-cytopathic) after transfection of dicistronic expression plasmids into BHK cells containing wild-type and mutagenized BVDV-sequences[15,16]. We show that the BVDV IRES irrespectively of the pathogenic properties of the individual strains is a strong ribosomal entry site. We provide evidence that the BVDV strain SD-1 IRES translational efficiency is increased by BVDV N-terminal non-coding region and contains a RNA pseudoknot structure that is indispensable for IRES function. These features exhibit remarkable similarity to the IRES of HCV and are not common with the IRESes of picornaviruses represented by the cardioviruses or enteroviruses, emphasizing that BVDV SD-1 IRES matches well into this distinct group of internal ribosomal landing pads.

## Results

### Strength of BVDV strains SD-1 and NADL IRES in BHK cells

**Figure 1:**
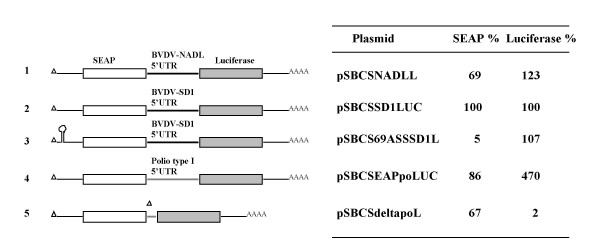
**BVDV-RNA translation in mammalian cells is mediated by a cap-independent, internal initiation of translation**. Left panel: Schematic representation of the mRNA arising from the expression plasmids stably transfected into BHK cells. Triangle, cap structure; solid line, intercistronic region with 5' UTRs of BVDV strain NADL, BVDV strain SD-1, or poliovirus type I. White box, SEAP reporter gene (secreted form of the alkaline phosphatase human placenta); grey box, luciferase reporter gene. The stem-loop structure in construct 3 has a calculated stability of about -73 kcal/mol. Right panel: Relative SEAP and luciferase expression values, levelled out to the specific mRNA content after Northern Blot quantification using a phosphorimager (see Materials and Methods).

Transfection of dicistronic vectors is a means to identify sequences responsible for cap-independent, internal initiation of translation. If the region in question is an IRES, translation of the second cistron may occur via internal entry of ribosomes in contrast to re-initiation which is possible only under very specific conditions. To exclude re-initiation, stable stem-loops may be included in the UTR preceding the first cistron to inhibit the scanning of 43S ribosomal complex that entered via the cap-structure. We have stably transfected into BHK cells expression plasmids pSBCSNADLLUC and pSBCSSD1LUC which carry the genes for the secreted form of the alkaline phosphatase (SEAP) and the firefly luciferase as reporters and the complete BVDV 5'UTR (NADL strain or SD-1-strain, respectively) as intercistronic region. For evaluation of the BVDV IRES strength pSBCSdeltapoLUC and pSBC-SEAP-Polio-LUC were chosen which are similar dicistronic devoid of any IRES or containing the poliovirus type IRES which is a strong mediator of internal initiation of translation [17]. In these and following experiments Northern Blot analyses revealed that the dicistronic mRNAs are of the expected length and no degradation products were observed which may result from endonucleolytic RNA cleavage or transcription by a cryptic promoter (data not shown). In addition, steady state mRNA levels were determined by phosphorimager quantification to account for differences due to variance in mRNA stability. Values shown are average values achieved from several experiments. Luciferase expression levels suggest that the 5'-UTRs of both BVDV strains mediate efficient translation of a second cistron in a dicistronic mRNA (Fig. 1, construct 1 and 2) irrespective of the cytopathic potential of the individual strains. In BHK cells the translation efficiency mediated by the BVDV-5'UTRs is approximately fivefold lower compared to the poliovirus type I IRES directed translation (compare constructs 1 and 2 with 4). To differentiate further cap-independent, internal initiation of translation from re-initiation of ribosomes after they completed translation of the first cistron pSBCSSD169L was constructed. pSBCSSD169L is a derivative of plasmid pSBCSSD1LUC and exhibits a stable hairpin-structure into the 5' UTR upstream of the first open reading frame. The calculated stability of the stem-loop of ΔG = -73 kcal/mol should be sufficient to interfere with cap-mediated, and ribosomal scanning-dependent translation [18]. The hairpin structure reduced SEAP translation 20 fold without affecting translation of the downstream luciferase cistron (Fig. 1, construct 3 and 2). Thus, internal initiation rather than re-initiation accounts for cistron 2 translation. Taken together the data show that both BVDV 5' UTRs represent IRES elements of medium strength and that differences of the individual strains in e.g. cytopathic or growth properties are not correlated to variances in efficiency of the initiation of translation in our test system.

### Deletion mutagenesis of BVDV SD-1 5' UTR

**Figure 2:**
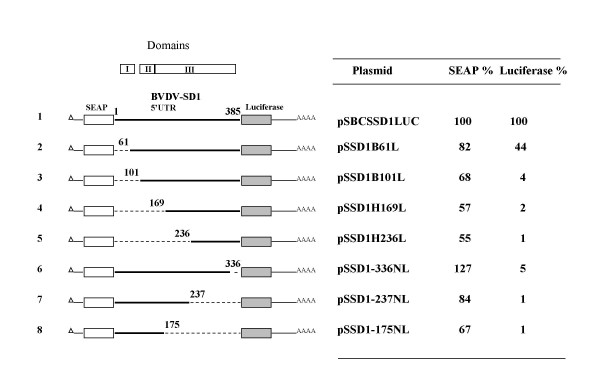
**Deletion mutagenesis of the BVDV SD-1 5' UTR**. Relative translation efficiency in BHK cells stably transfected with dicistronic expression plasmids carrying 5' and 3' deletions in the 5' UTR. The SEAP and luciferase values are normalized to specific mRNA levels. Domains depicted in Fig. 3 are indicated above the schematic representations of the expression plasmids.

**Figure 3:**
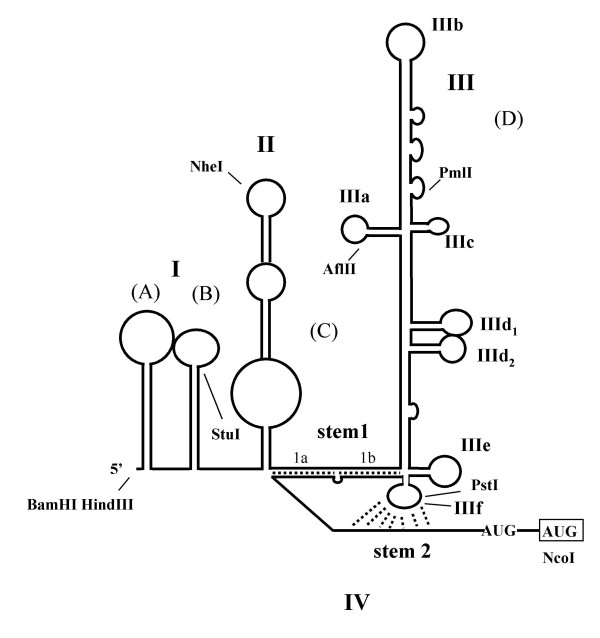
**Proposed RNA secondary structure of the BVDV (strain SD-1) 5' UTR**. The map was adapted from computer-predicted structures published by Deng and Brock [19]. The domain denomination by Deng and Brock makes use of uppercase letters. The nomenclature used in Brown et al. is indicated by roman numbers [20]. Two out of the seven AUGs in the BVDV leader are shown, the AUG used for initiation of translation is boxed. The putative RNA pseudoknot interaction is depicted by dashed lines. Arrows indicate the position of restriction enzymes used to construct the deletion mutants.

The borders of the IRES element of pathogenic BVDV strain NADL have been determined previously [1,2]. To delineate the IRES boundaries in the related, IRES of non-pathogenic BVDV SD-1, a series of dicistronic plasmids carrying SD-1 5'UTRs with sequential deletions in the 5' and 3' direction were transfected into BHK cells (Fig. 2). Luciferase translation decreased twofold in the construct devoid of the 5' terminal 61 bases and dropped dramatically in all further 5'-3' deleted mutants (Fig. 2. constructs 2–5). Similar low levels of luciferase expression were found in all experiments with 5' UTRs carrying deletions extending from the initiator AUG in the upstream direction (Fig. 2, constructs 6–8). The data demonstrate that bases 61–385 of the BVDV 5' UTR are essential for efficient translation and that the 5' terminus of the UTR contributes only marginally to translation efficiency. The region encompasses about 80% of the 385 nt BVDV 5' non-coding region suggesting that long range RNA interactions may be involved in internal landing of ribosomes. The 5' terminus of the BVDV SD-1 5' UTR contributes only marginally to translation efficiency suggesting that domain I (stem loops A and B) [19,20] are dispensable. In contrast, stem-loops II and III (C and D) are required for the initiation process (Fig. 3). The data are in agreement with results from investigations of the BVDV strain NADL IRES published earlier [2].

### The BVDV coding region contributes to translation efficiency mediated by the BVDV SD-1 IRES

**Figure 4:**
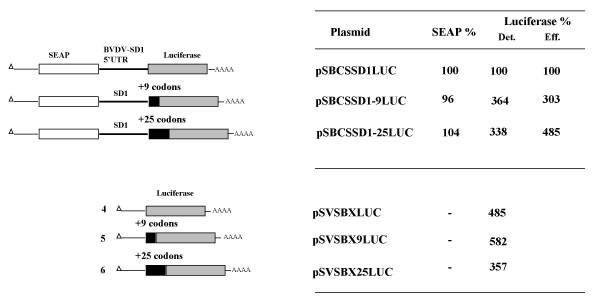
**The influence of BVDV coding sequences on IRES mediated translation**. Left panel: Schematic drawing of dicistronic (1–3) and monocistronic (4–6) plasmids carrying the complete BVDV-5' UTR or the 5' UTR plus 5' proximal BVDV SD-1 coding regions. Right panel: Relative translation efficiency of SEAP and luciferase reporter genes of the respective dicistronic or monocistronic plasmids in stably transfected BHK cells. The SEAP and luciferase values are normalized to specific mRNA levels. Luc (Eff): Luciferase values exhibited by the moncistronic constructs were taken into account to calculate the effect of the inclusion of coding region on IRES mediated translation. The AUG context at position +4 (G) and +5 (A) of the wild-type luciferase construct and its fusion mutants is identical and optimal and should not give rise altered translational efficiency [60]. The data shown are average values derived from four independent experiments. Addition of 9 or 25 codons of the BVDV aminoterminus (black box) to the SD-1 5' UTR results in 3 to 4.8 fold increase of translation efficiency when the effects of N-terminal extension of luciferase in monocistronic constructs on luciferase stability/activity were considered.

The involvement of coding sequences immediately down-stream of the 5'UTR has been documented for initiation of translation of pestivirus RNA (BVDV NADL strain, CSFV) and also HCV [2,10,14,21,22]. A role for coding regions was excluded in cardiovirus IRES mediated translation [11], but has been reported previously for the IRES element of hepatitis A virus (HAV), a picornavirus [23]. To investigate whether the SD-1 IRES extends into the BVDV coding region mono- and dicistronic expression plasmids were stably transfected into BHK cells carrying the complete BVDV SD-1 5' UTR, or the UTR extended by either 27 or 75 bases into the contiguous protein coding region were constructed (Fig. 4). The coding sequences of the BVDV N^pro ^were in-frame with the downstream luciferase reporter and resulted in N-terminal extension of the luciferase protein by 9 and 25 amino acids, respectively. First, to determine the effect on the luciferase reporter of these added amino acid residues, monocistronic expression plasmids 4, 5 and 6 were compared. Construct 4 is firefly luciferase expression vector, while expression plasmids 5 and 6 additionally harbored 9 and 25 codon in-frame fusion to the original firefly luciferase cDNA. Analysis of the stability of the luciferase mRNA revealed no differences among these constructs (data not shown). Protein expression, as measured by luciferase activity, also appeared only slightly affected by the addition of either 9 or 25 amino acids derived from the BVDV N^pro ^protein in these monocistronic constructs (Fig. 4) N-terminal fusion of luciferase with 9 amino acids of the BVDV capsid N-terminus resulted in 1.2 fold increased activity, presumably due to an increase in luciferase stability [24], inclusion of 25 amino acids of N^pro ^reduced luciferase activity 1.4 fold. These alterations in activity of luciferase-fusions in the monocistronic constructs was taken into account to calculate the final enhancement of BVDV coding sequences out of the data a for luciferase translation in the dicistronic constructs of Fig. 4. Thus, in the dicistronic constructs 1, 2 and 3 the addition of 27 or 75 nucleotides of BVDV aminoterminal coding region resulted in an 3 fold or 4.8 fold increase in translation efficiency. Inversion of the 25 residue N^Pro ^sequence in construct 3 resulted in a 16 fold decrease of second cistron translation compared to construct 3 (data not shown). The results demonstrate that the BVDV IRES expands into the BVDV coding region and that sequences immediately downstream of the BVDV initiator AUG contribute to the efficiency of internal initiation in pestivirus BVDV strain SD-1. Taken together with previous observations with BVDV NADL, CSFV and HCV, one may speculate that coding region involvement is unique if compared to other viral and cellular IRES elements, but is a 'common' feature in related pestiviruses and hepaciviruses.

### The importance of both stems of the pseudoknot structure

**Figure 5:**
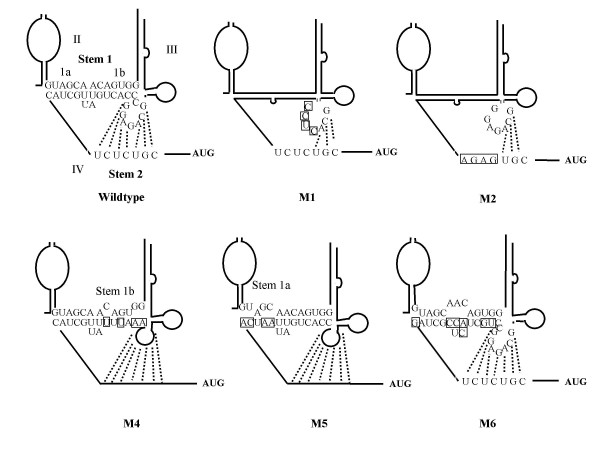
**Mutagenesis of stem 1 and stem 2 of the proposed pseudoknot structure in the BVDV SD-1 5' UTR**. A. Schematic drawing of wild-type plasmids and pseudoknot mutants. Plasmid pSBCSSD1-9LUC (Fig. 4) was used as basic plasmid for construction of the pseudoknot mutants. Altered nucleotides are boxed. In mutants M1 and M2 stem 2 base-pairing is disturbed, in M4, M5, and M6 the stem 1 complementarity is impaired.

**Figure 6:**
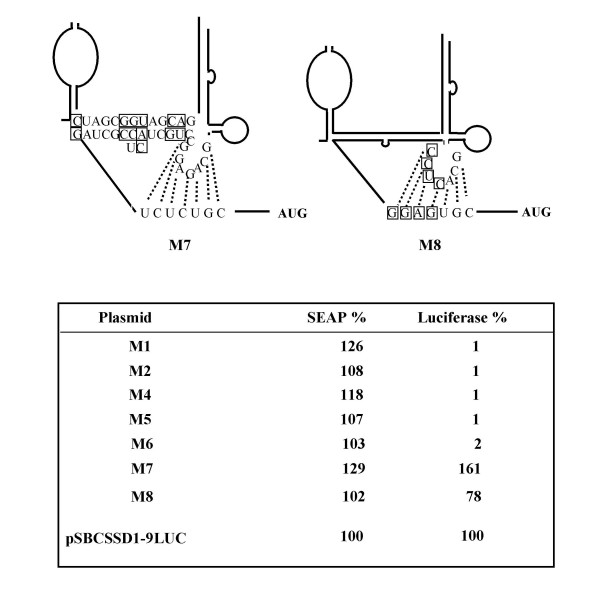
**Compensatory Mutations and expression levels**. Top: In M7 the M6 mutations introduced in stem 1 are compensated, restoring stem 1 integrity and giving rise to a sequence resembling BVDV genotype 2. In M8 nucleotide exchanges were made to compensate for mutations introduced into stem 2 of mutant plasmids M1. Bottom: Relative SEAP and luciferase expression values normalized to specific mRNA levels in BHK cells stably transfected with wild-type and pseudoknot mutant plasmids depicted in figures 5 and 6.

**Figure 7:**
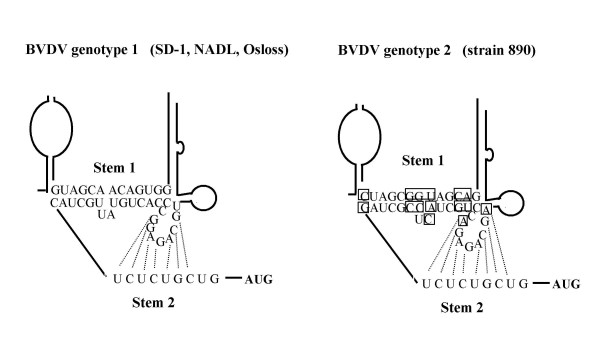
**The predicted pseudoknots of BVDV genotype 1 and 2**. Stem interactions are conserved within the BVDV genotypes. Divergent nucleotides in genotype 2 pseudoknot are indicated by boxes. Note that nucleotide substitutions in one strand of stem 1 of BVDV genotype 2 are compensated by complementary mutations in the opposite strand so that stem 1 and stem 2 interactions are highly conserved.

Based on the predicted RNA secondary structural models in HCV and pestiviruses Le et al. searched for tertiary interactions and identified a pseudoknot region immediately upstream of the initiator AUG in HCV and in pestiviruses [25]. Subsequently, the physical presence of the predicted pseudoknot structure in HCV was demonstrated by biochemical analysis, and evidence for the functional role of the pseudoknot in HCV internal ribosome entry was provided by mutagenesis experiments for HCV and CSFV [3,12,13]. In contrast to HCV in all pestiviruses stem 1 of the pseudoknot is bipartite and carries an intervening loop between stem 1a and stem 1b. The length of the stem 1 a and b in BVDV are 6 and 7 bp, respectively. To investigate whether the proposed pseudoknot structure in the BVDV 5' UTR is part of the BVDV translational strategy, we determined reporter gene expression after transfection of dicistronic plasmids carrying mutations in the putative pseudoknot structure (Fig. 5, Fig. 6). Mutants M1 and M2 carry contiguous substitutions in bases 341–344 (upper strand) and 367–370 (lower strand) of stem 2, respectively, and interfere with formation of pseudoknot stem 2 (Fig. 5). Mutants M5 and M4 exhibit non-contiguous substitutions in the left (stem 1a) or right (stem 1b). M6 addionally carries substitution in the central portion (around the 'bubble') of stem I. Mutations M4, M5, M6 impair the formation of stem 1. Luciferase expression levels revealed that all mutations which perturb the structure of stem 1 or stem 2 dramatically reduced the ability of the 5' UTR to mediate internal initiation of translation. All pseudoknot mutants disrupting parts of the stem structure were translationally inactive, irrespectively of the strand of the stem in which the mutation was introduced (Fig. 6). In mutant M7 disrupting base changes of mutant M6 were repaired by introducing complementary bases in the opposite strand (Fig. 6). Interestingly, the repaired stem resembles the sequence found in genotype 2 BVDV 5'UTR (see Fig. 7). The compensatory mutant not only restored IRES activity, but slightly enhanced translation efficiency, thereby demonstrating that intact pseudoknot tertiary structure is of importance for IRES mediated translation. To confirm the importance of stem 2 integrity pseudoknot mutant M8 was constructed which restored base pairing and compensated for mutations inserted into stem 2 in mutant M1. Again, translational activity, which dropped down to 1% of the wt SD-1 IRES in mutant M1, could be restored in compensatory mutant M2 to 78% of wt level. In summary, the results from mutational analysis of stem 1 and stem 2 of the putative pseudoknot indicate the relevance of this region of tertiary structure for BVDV translation.

### Strength of the BVDV strain SD-1 IRES in cell lines of human and murine origin

To evaluate the translational efficiencies in cell lines of different origin, we transfected dicistronic expression vectors containing the SD-1 5'UTR or the poliovirus IRES into cell lines of mouse and human origin [see additional file 1]. In some experiments a vector containing the SD-1 IRES extended by 9 amino acids of the coding region was also used. Furthermore, control vectors devoid of an IRES in the intercistronic region or carrying the inhibitory stem-loop in the 5'UTR of the dicistronic mRNA were included into the experiments. The cell lines were derived from different tissues and include cancer cells like glioma and neuroblastoma (brain), myeloma, erythroleukemia (blood), hepatoma (liver), carcinoma (cervix) and a kidney cell line often used for transient protein production. Interestingly, in all murine cell lines investigated the BVDV SD-1 IRES exhibited higher expression levels than the poliovirus IRES. In human cell lines – with the exception of a cervix carcinoma cell line – the poliovirus IRES mediated higher luciferase expression than the SD-1 5'UTR. Interestingly, in HeLa cervix carcinoma the SD-1 5' UTR mediated 2.4 fold higher second cistron expression than the poliovirus IRES. Including the extension into the coding region into the SD-1 IRES resulted in 1.5–3 fold increase in translational efficiencies irrespective whether the cell line is of human or mouse origin. As expected, second cistron expression was blocked, when the expression vector contained an intercistronic region devoid of IRES activity. Incorporation of an inhibitory stem-loop in front of cistron 1, abolished cistron 1 expression as expected, but also effected cistron 2 expression slightly but to a certain extent. Taken together, SD-1 IRES meditates higher expression levels in cell lines of murine origin compared to the poliovirus type I IRES, which may have its molecular basis in the equipment of the cell with specific factors necessary for translation mediated by the individual IRES.

## Discussion

Translation of the BVDV RNA strain NADL occurs via internal initiation of translation [1,2]. We confirmed and extended these data by transfection experiments with dicistronic plasmids using the strain NADL and SD-1 5'-UTRs as intergenic regions. Insertion of an inhibitory stem-loop structure in the 5' UTR of the dicistronic mRNA lead to severe reduction of cistron 1 translation, but had no effect on BVDV5' UTR mediated translation of cistron 2. The insensitivity of downstream cistron translation to the inhibition of scanning dependent translation is a strong indicator of an internal ribosomal entry versus a re-initiation of ribosomes after translation of an upstream open reading frame.

A central part of our study included the determination of the borders of the BVDV IRES of the non-cytopathic SD-1 strain. The BVDV 5' UTR is 385 nucleotides in length. We found, that the BVDV IRES encompasses about 80% of the 5' UTR. The 5' proximal 20% of the BVDV leader contributes only marginally to SD-1 IRES function, which is in agreement with results from deletion analysis of the NADL 5' UTR and hybrid arrest translation experiments performed earlier [1,2] However, deletions further downstream or deletions in the opposite, upstream direction starting from the authentic translational initiation site severely inhibited BVDV IRES function. The results indicate that an overall higher order structure formed by stem-loop regions II (C) and III (D) (Fig. 3, [19,20]) as well as the region between region III and the initiator AUG which contribute to the pseudoknot structure are important and must be preserved to guarantee IRES function. Our results from experiments with the strain SD-1 5'UTR are in agreement with earlier investigation of the related strain NADL IRES. Previous mapping experiments using an incomplete BVDV leader missing the 5' proximal 28 nt have demonstrated that partial removal of domain III (D) by deletion of bases 173–236 resulted in a 3 fold decrease in IRES mediated translation in transfected BHK cells [1]. *In vitro *experiments using hybrid arrest translation identified a region 154–261 within the domain III (D) structure to be important for BVDV protein synthesis [1]. Fine mapping of the BVDV NADL IRES revealed that stem-loops Ia and Ib were dispensable for efficient translation and the hairpin end of IIIb and stem-loop IIIe were only partially required. In contrast, deletions in domains II, IIIa, IIIc and IIId caused nearly 10 fold decrease in BVDV NADL IRES in vivo activity, stressing the importance of these regions for translation [2]. The results concerning the 5' UTR boundaries of the BVDV IRES parallel the results reported for the mutational analysis of the closely related HCV 5' UTR[4,5,26] and pestiviral CSFV IRES [3] which indicate that the HCV and CSFV IRESes include almost the entire 5' non-coding region emphasizing the close relationship of HCV, CSFV and BVDV 5' UTR in structure and function.

Remarkably, the efficiency of translational initiation from pestivirus and HCV IRESes and also HAV is influenced profoundly by the nature of the 5' proximal coding region, which suggested an IRES extension into the coding region [10,14,21-23,27]. While the 'IRES extension' into the coding regions has been mapped in detail for HAV, HCV and CSFV [10,14,23], the coding region requirement has not been investigated in detail in BVDV. Chon and co-workers included a 515 nt ORF region as extension into their investigation of the BVDV NADL IRES 3'delimitation. Deletion of the long coding region reduced IRES activity to 79%, which supported the idea that the NADL IRES extends into the coding region and that N^pro ^coding region contributes to IRES efficiency, however only marginally [2]. A remarkable result of our investigation was achieved when we extended the BVDV SD-1 IRES in our experiments by short coding regions following the start AUG of the polyprotein. To circumvent problems that may be related with stable secondary structures immediately downstream of the AUG initiation codon, firefly luciferase was used as a reporter gene in the translation of the second cistron [28]. As expected for the related BVDV strain the IRES mediated translation was enhanced by the polyprotein coding region. However, in contrast to the low enhancement in case of the NADL-N^Pro ^addition reported earlier [2] we found a 3 to 4.8 fold enhancement of translation efficiency after addition of 27 or 75 nt of the N^pro ^coding region to the 5'UTR. Additional support for the importance of the sequences immediately downstream of the initiating AUG is provided by the comparison of the 5' terminal coding region of various BVDV isolates. Due to the high mutation rates of RNA a considerable variation in the wobble position of the BVDV sequences is expected [29]. However, the alignment of nucleic acids and protein sequences of 3 BVDV genotype I isolates (NADL, SD-1, Osloss) and one genotype 2 isolate (2–890) indicates low variation in the wobble position in the N-terminal coding sequence. 13 out of 16 codons are totally conserved with respect to nucleic acids sequence in the first 16 codons of the BVDV polyprotein (data not shown). This fairly conserved region is followed by an area of high divergence, only 2 out of the following 16 codons remain the same in all four pestivirus strains. Interestingly, a similar conservation scheme is observed in primary structure alignments of CSFV strains (Brescia and Alfort), where 14 out of the 16 aminoterminal codons were conserved within these two strains while divergence appeared after codon 15 (data not shown). This notion correlates with the findings that 17 codons of the N-terminal region are required for CSFV IRES translational enhancement [14], while shortening to 12 codons resulted only in 66% of translational efficiency. Theses findings suggest a strong selective pressure on preservation of the nucleic acid sequence suggesting an importance of the region for internal initiation of translation rather than a constraint for amino acid preservation.

Support for our notion that the 5' proximal N^Pro ^region is important for translational initiation came from experiments mapping the 40 S binding segment in BVDV RNA. Similar to HCV, the BVDV IRES is able to bind 40S ribosomal subunits directly without the need of initiation factors. The BVDV RNA generates toeprints (primer extension inhibition) that indicate interaction at position U361 of the pseudoknot in the 5' UTR and positions 10–12, +15 to +17 and +19 with respect to the initiator AUG in the NPro coding region [30]. Interestingly, similar to the situation in CSFV the interaction seems to be very sensitive to secondary structures immediately downstream to the initiator AUG, which resembles the situation in prokaryotic systems [14,28,31]. Myers et al. argued that absence structural constraints, rather than binding of a cellular factor is responsible for N^Pro ^augmentation or BVDV translation[31]. The importance of BVDV N-terminal coding region in viral replication was demonstrated in DI particles where 'subgenomic' RNAs with internal in-frame deletions derived from mutant BVDV viruses are observed. Interestingly, the N-terminal 28 amino acids of the N^Pro ^coding region were retained in 11 of the mutant viruses. In an attempt to construct BVDV replicons constructs failed with reporter genes directly fused to the BVDV 5' UTR but mutants could be rescued when 12 to 84 nucleotides of N^Pro ^N-terminal sequences were added [32-34].

The BVDV SD-1 coding region contributes moderately but distinctly to enhance initiation of translation. Presently, it is not clear whether a low degree of secondary structure, a cellular protein that binds to the proximal region downstream of the AUG codon, or other factors contribute to the effect observed in our investigation. The contribution of coding region to translational initiation represents a complex issue, reflected by the fact that some researchers observed the effect in HCV and pestiviruses [2,10,14,21,22,27] and others did not [4,5,26,28]. In HCV and pestivirus translation 40 S ribosomes bind directly to the viral RNA without the need of additional factors [30,35,36]. Due to the absence of an RNA helicase (as present in picornavirus initiation of translation), the 40S ribosomal subunit binding is impaired by stem-loop structures in the vicinity of the initiator AUG in HCV and pestivirus translation [14,28,31]. As mutants with less stable secondary structure in the AUG proximal coding region give rise to an increase of translation, 40 S binding seems to be sensitive to stem-loops downstream of the initiator. Thus, a low degree of secondary structure largely, but not exclusively, contributes to coding region enhancement of translation. Interestingly, in a recent report Kim et al. identified the cellular RNA binding protein NSAP1 that modulates HCV IRES-mediated translation. NSAP1 binds to the run of A residues in the region of low secondary structure in the HCV N-terminus, identified as part of the coding region which augments HCV IRES mediated translation. In a series of experiments they showed that the cellular protein is crucial for increase of the translational efficiency of the HCV IRES [37]. The involvement of coding region in IRES mediated translation of viral RNAs has been demonstrated recently in two other cases, which corroborate the importance of coding regions in internal initiation of translation. Garlapati et al. showed that in Giardiavirus (GLV), a double-stranded RNA plant virus of the *totiviridae *family, the IRES extends to both sides of the AUG initiator codon [38]. Interestingly, a stable stem-loop in the vicinity downstream of the initiator AUG does not interfere with GLV translation. Surprisingly, Herbetreau and co-workers found, the HIV-2 RNA contains a new type of IRES which is located within the coding region [39].

Another interesting result of our investigation was the finding that a pseudoknot structure postulated by computational RNA folding actually is involved in BVDV IRES function. From the genetic data presented we conclude that the putative pseudoknot in the BVDV SD-1 5'UTR is an important element for IRES function. Strikingly, alterations in the termini of each half (1a, 1b) or the center of stem 1 as well as mutation of 4 consecutive bases in each strand in the centre of stem 2 abrogated IRES function. However, IRES function could be reconstituted through construction of mutants (M7, M8) compensating the nucleotide exchanges in the secondary structure of stem 1 or 2 (mutants M6, M1). This strongly suggests tertiary structure requirements in IRES function. Pseudoknot structures play a role in ribosomal frameshifting, cleavage in group introns and hepatitis delta virus, protein recognition for translational regulation and autoregulation [40]. The involvement of a pseudoknot in the internal initiation of translation was shown previously for the HCV IRES [12,13] by biochemical and genetic methods to prove the presence and the function of the pseudoknot. A potential pseudoknot was computed in BVDV 5' UTRs by thermodynamical, phylogenetic and statistical methods. Thermodynamic calculations based on different programs (EFFOLD, SEGFOLD, RNAKNOT) showed that this tertiary structure represents a highly conserved feature among different pestiviruses and HCV [13,25,41-43]. Previously, Rijnbrand et al (1997) and Fletcher and Jackson (2002) provided genetic evidence for pseudoknot involvement in CSFV RNA translation [3,44]. Rijnbrand et al. showed that mutants that lost the ability to base pair in stem II of the pseudoknot were translationally inactive in mammalian cells and translation to wild-type level could be restored by the introduction of compensatory base changes in stem II. Fletcher and Jackson confirmed the previous findings and extended their analysis to pseudoknot stem 1a and the loop structure between the two stems of the pseudoknot. They demonstrated the importance of stem 1 integrity and showed that the length of the loop between the two stems and clustered A residues were crucial for CSFV IRES activity.

Due to differences in primary structure and immunological properties, BVDV strains are divided into two genotypes. Genotype 1 encompasses the classical BVDV isoloates (NADL, SD-1, Osloss) while genotype 2 refers to later described isolates (e.g. 2–890) [45,46]. Interestingly, the primary structure of the pseudoknot stems is conserved within the BVDV genotype 1, but base substitutions were observed in comparison to the pseudoknot stems of the BVDV genotype 2 (Fig. 7). BVDV pseudoknot primary structure of genotype 1 and the genotype 2 differ in 13 out of 23 nts in stem 1 and 2 nts in stem 2. Interestingly, mutations in the opposite strand for stem 1 compensate for alterations of the complementary strand in genotype 2, and the G-A change at pos. 352 and C-A change at pos. 359 in BVDV2 increase the stability and the length of stem 2. This appearance of a natural compensation of primary structure divergence in order to conserve the respective higher order structure strongly argues for the importance of the pseudoknot for both genotypes. Presently, the role of the pseudoknot in BVDV translational initiation is not known. It is tempting to speculate that it supports IRES basal region III in binding of 40 S ribosome or acts in concert with other IRES domains in AUG positioning, as has been suggested recently for the HCV IRES based on modelling data [47-49].

Taken together, the BVDV SD-1 IRES shares features previously reported for the BVDV NADL, CSFV and HCV IRESes. The most prominent characteristics are the IRES length of about 330–380 nucleotides, the involvement of a pseudoknot structure, the participation of coding sequences in translation efficiency and a direct ribosome binding and initiation mechanism without the requirement of additional factors [30]. Due to the differences to type I and type II IRES elements, hepacivirus HCV and pestiviruses IRESes represent a distinct group of viral IRES elements, denominated type III or, more recently, HP IRESes [50]. Interestingly, a subset of viruses within the picornavirus family has been identified recently, which resembles the hepacivirus and pestivirus IRES elements and which may join this interesting group in the future [50].

## Conclusion

Translational efficiency of the IRES of the non-cytopathic BVDV SD-1 is increased by the non-coding region immediately downstream of the AUG initiator codon and is higher in murine cell lines compared to cell lines of human origin. The putative pseudoknot found in the IRES of the non-cytopathic BVDV SD-1 strains represents an important structural element in translation of the viral RNA. Since the BVDV pseudoknot region is crucial for polyprotein translation, it may represent a feasible target for blocking viral replication, e.g. by RNA interference as it has been demonstrated for HCV [51].

## Methods

### Cells and cell culture

BHK21 (ATCC CCL10), HeLa (ATCC CCL2), HEK 293 (ATCC CRL1573), AS-30D rat hepatoma (DSM ACC208), SNB19 human glioma (DSM ACC325), LN405 human glioma (DSM ACC189) cells were maintained in DMEM supplemented with 10% fetal calf serum, 100 μg/ml streptomycin and 100 U/ml ampicillin. For cultivation of C6 rat glioma (a kind gift of Bernd Hamprecht, Univ. Tuebingen) antibiotics were omitted. Sp2/0 mouse myeloma (a gift of Uli Weidle, Roche Diagnostics), HepG2 cells (ATCC HB8065) as well as K562 human erythroleukemia (DSM ACC10) were propagated in RPMI medium supplemented with 10% calf serum. For HGBM1 human glioma (via H. Weich derived from Dr. Megyasi, Harvard Medical School) cultivation in a 1:1 mixture of RPMI/DME and 10% FCS was applied. HT1080 human fibrosarcoma (CCL 121) were cultivated in MEM with Earle's BSS and essential amino acids and 10% FBS and. If not otherwise indicated cell lines originated from ATCC or DSMZ (German collection of micro organisms and cell culture).

### Plasmids and plasmid construction

Recombinant DNA technologies were performed by standard procedures [52].

The construction of pSBCSEAPpoLUC and pSBCSdeltapoL is described elsewhere [17]. pAG60 [53] and pSV2PAC [54] mediate G418 and puromycin resistance to mammalian cells, respectively.

pST7-1568A13 [16] harbors the 5' UTR and a part of the coding region of the BVDV strain SD-1 genome. pT7 5'p20 encodes the 5' UTR and a portion of the coding region of BVDV strain NADL[15]. pmβActin (Stratagene) harbors the cDNA of the mouse β-actin gene.

pSVSBXLUC is a eukaryotic expression vector harboring the SV40 early promoter, single cloning sites for SacII, BamHI, XhoI, NcoI, a firefly luciferase cDNA and the luciferase polyadenylation signal. The luciferase translational start codon is the AUG of the NcoI site.

pSBC2SEAP is derived from pSBC2 [17] and harbors the SEAP coding region under control of the SV40 early promoter.

Fragments containing the complete 5' UTRs of BVDV strain NADL and SD-1 were generated by PCR from plasmids pT7 5'p20 [15]and pST7-1568A13 [16]. The 5' primers were designed to supply missing nucleotides in the extreme 5' part of the untranslated region. To facilitate cloning, the 5' primers carried a BamHI and HindIII site, while the 3' primers were equipped with a NcoI site. For construction of pSBCNADL-A and pSBCNADL-AUG 3' primers were used which carried the respective mutations in the central part of the oligonucleotide. The PCR fragments were cut with BamHI and NcoI and cloned into BamHI/NcoI linearized pSVSBXLUC. The resulting monocistronic plasmids pSVNADLLUC, pSVSD1LUC pSVSD1ALUC and pSVSD1AUGLUC were cleaved with HindIII and the fragment containing the 5'UTR, luciferase cDNA and luciferase pA was inserted into HindIII linearized pSBC2SEAP to give rise to the dicistronic plasmids pSBCNADLL, pSBCSSD1LUC, pSBCNADL-A and pSBCNADL-AUG, respectively.

pSBCS69ASSSD1L is derived from pSBCSSD1LUC by insertion of NotI terminated, 'stemloop' oligonucleotides 69 nt in length into the single Not I site 5' to the SEAP start codon.

For the construction of dicistronic deletion mutants monocistronic plasmids carrying the deleted forms of the SD1–5' UTR were constructed by digestion of monocistronic pSVSD1LUC with NcoI and PstI, PmlI or AflII (deletion at the 3' end of the leader), polishing of the ends with T4DNA ligase or Klenow and religation. The respective dicistronic plasmids were constructed by ligation of HindIII fragments derived of the monocistronic plasmids and carrying the mutated BVDV-5'UTR, the luciferase cDNA and pA into HindIII linearised pSBC2SEAP as described above. 5' deleted leader mutants pSSD1H236L and pSSD1H169L were derived from partial digestion of dicistronic plasmid pSBCSSD1LUC with HindIII followed by cleavage with PmlI or AflII and religation of the long fragment.

pSSD1B61L: StuI/BamHI cleaved pSVSD1LUC was religated after end polishing and the BamHI/HindIII fragment carrying the deleted BVDV-5'UTR and the luciferase cDNA was ligated into HindIII cleaved pSBC2SEAP after fill in of overlapping ends.

pSD1B101L: The 285 bp NheI filled in/NcoI fragment was ligated into BamHI filled in/NcoI digested pSVSBXLUC. The 5' BVDV-UTR-Luciferase fragment was isolated from the resulting plasmid after BamHI/HindIII digestion and end polishing and was ligated into HindIII filled in pSBC2SEAP.

pSVSBX9LUC and pSVSBX25LUC: Oligonucleotides with overlapping NcoI sites coding for the terminal 9 and 25 amino acids of the p20 protein were ligated to NcoI digested pSVSBXLUC.

pSBCSSD1-25LUC and pSBCSSD1-9LUC: The 5'UTR luciferase HindIII fragment of pSVSBX9LUC or pSVSBX25LUC was cloned into HindIII linearised pSBC2SEAP.

Pseudoknot mutants: Oligonucleotides carrying mutations in the pseudoknot as shown in Fig. 5 and PstI or NcoI ends substituted for the 48 bp PstI/NcoI fragment of pSVSBX9LUC. The HindIII fragments carrying mutated 5'UTRs and the luciferase cDNA were cloned into HindIII linearised pSBC2SEAP.

The integrity of plasmids derived from PCR cloning and oligonucleotide cloning was confirmed by dideoxy nucleotide sequencing using the Sequenase sequencing kit (USB).

### Enzymes

Restriction enzymes, DNA polymerase and T4 DNA ligase were purchased from New England Biolabs and Roche Diagnostics.

### Oligonucleotides

Oligonucleotides were synthesized on 380B or 394 synthesizers (Applied Biosystems) and purified by OPC affinity chromatography (Perkin Elmer).

### Transfection

Plasmids were co-transfected as supercoiled DNAs either using the calcium phosphate co-precipitation as described [55] or lipofectamine. Transient expression experiments were analysed 2 d after gene transfer. For stable transfection of BHK cells, depending on the co-transferred selection marker, selection with either 700 μg/ml G418 or 5 μg/ml puromycin was applied 48 h after transfection. In the case of G418 selection cells were split 1:3. Clones arising 8–12 d after selection were counted and pooled.

### Reporter gene assays

The colorimetric SEAP assay was performed as described in [56], the more sensitive luminometric SEAP determination was done as recommended by the supplier (Phosphalight, Tropix). Luciferase activity was determined after repeated thawing and freezing of cells according to the protocol of de Wet et al. [57] as 'flash type' assay in a Lumat LB 9501 (Berthold, Germany). The 'glow type' assay (LucLite, Canberra-Packard) was used for processing large number of samples in microtiter format in a Microlumat LB96 P (Berthold, Germany).

Parallel measurements guaranteed reliability of the individual assay types. SEAP or luciferase productivities were calculated in light units/10^6 ^cells a day. To account for differences in mRNA levels due to variation in mRNA stability, these values were adjusted to the specific mRNA levels using the data obtained for steady state mRNA concentration (see following chapter). The final translation efficiency of each cistron is given in%, the values of the dicistronic construct carrying wild-type SD1 5' UTR are arbitrarily set to 100%. Results represent average values from multiple, independent transfection series.

### RNA analysis

polyA^+ ^RNA was isolated from 10^6 ^cells using the mRNA DIRECT kit (Dynal, Norway) employing oligo dT-conjugated magnetic beads, separated on formaldehyde gels and blotted onto nylon membranes (Biodyne) according to standard procedures [52]. RNAs were hybridized with ^32^P labeled luciferase (or SEAP) cDNA and washing of the blots was performed under high-stringency conditions. Probing for reporter gene specific mRNA was followed by rehybridization with ^32^P labeled mouse β-actin cDNA to equalize differences in mRNA extraction. Radioactive signals of both hybridizations were analyzed and quantitated using a phosphorimager (Molecular Dynamics).

### Sequence alignment

Nucleotide and protein sequences were aligned using the pileup option of the GCG program package and published primary structures of BVDV NADL [15], SD-1 [16], Osloss [58], 2–890 [45], CP7 [59].

## Abbreviations

BVDV : bovine viral diarrhea virus; 

CSFV : classical swine fever virus; 

HCV : hepatitis C virus; 

IRES : internal ribosome entry site; 

SEAP : secreted alkaline phosphatase.

## Competing interests

The author(s) declare that they have no competing interests.

## Authors' contributions

MW conceived and supervised the study. LM performed the experiments. MW wrote the manuscript. All authors read and approved the final manuscript.

## Supplementary Material

Additional file 1 Translation efficiency mediated by the BVDV IRES in different cell lines. The data provide a comparison of BVDV SD-1 IRES strength in mouse and human cell lines derived from different tissues. Click here for file
