# Supplementary material for: The internal initiation of translation in bovine viral diarrhea virus RNA depends on the presence of an RNA pseudoknot upstream of the initiation codon
Source: Virol J. 2007 Nov 22;4:124. doi: 10.1186/1743-422X-4-124 (PMC2212637; doi:10.1186/1743-422X-4-124)
Supplement: Additional file 1 — Translation efficiency mediated by the BVDV IRES in different cell lines. The data provide a comparison of BVDV SD-1 IRES strength in mouse and human cell lines derived from different tissues. [file 1743-422X-4-124-S1.ppt]

## Slide 1
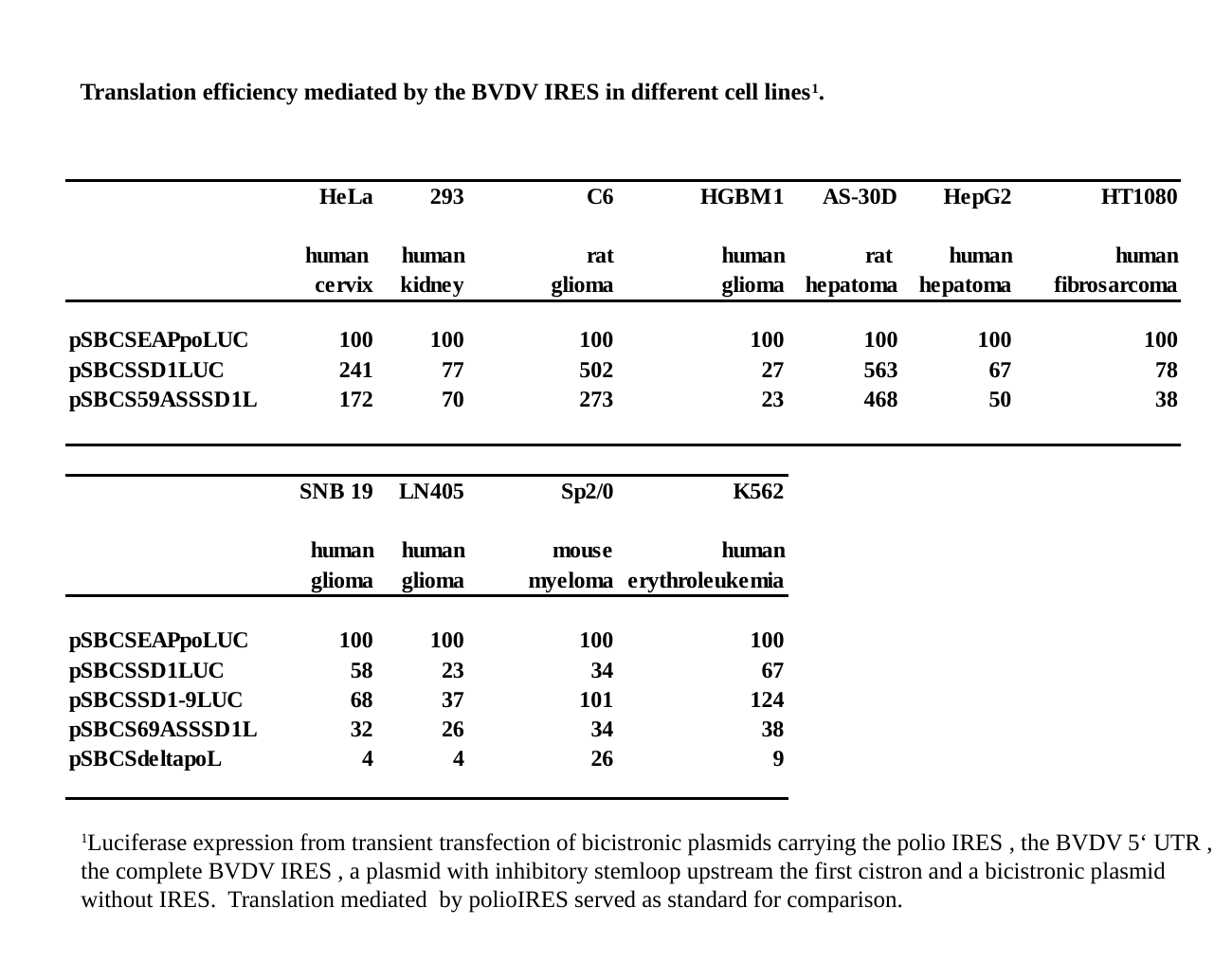

Translation efficiency mediated by the BVDV IRES in different cell lines1.
1Luciferase expression from transient transfection of bicistronic plasmids carrying the polio IRES , the BVDV 5‘ UTR ,
the complete BVDV IRES , a plasmid with inhibitory stemloop upstream the first cistron and a bicistronic plasmid
without IRES. Translation mediated by polioIRES served as standard for comparison.
